# Insights into microorganisms, associated factors, and the oral microbiome in infective endocarditis patients

**DOI:** 10.3389/froh.2024.1270492

**Published:** 2024-04-11

**Authors:** Ayden Ismail, Amieth Yogarajah, Joseph Luke Falconer, Rafal Dworakowski, Samuel Watson, Jonathan Breeze, Margaret Gunning, Habib Khan, Azhar Hussain, James P. Howard, Phoebe Cheong, Mira Shah, Luigi Nibali, Vanessa Sousa

**Affiliations:** ^1^ Faculty of Dentistry, Oral and Craniofacial Sciences, Centre for Host-Microbiome Interactions, King’s College London, London, United Kingdom; ^2^ Department of Anaesthesia, King’s College Hospital NHS Foundation Trust, London, United Kingdom; ^3^ Department of Periodontology, Guy’s and St Thomas’ NHS Foundation Trust, London, United Kingdom; ^4^ Department of Cardiology, King’s College Hospital NHS Foundation Trust, London, United Kingdom; ^5^ Department of Cardiothoracic Surgery, King’s College Hospital NHS Foundation Trust, London, United Kingdom; ^6^ National Heart and Lung Institute, Imperial College London, London, United Kingdom; ^7^ Imperial College Healthcare NHS Trust, London, United Kingdom

**Keywords:** endocarditis, oral microbiome, diagnostics, oral health, risk factor

## Abstract

**Introduction:**

Infective Endocarditis (IE) is a rare, life-threatening infection of the endocardium with multisystem effects. Culprit microorganisms derived from different niches circulate through the bloodstream and attach to the endocardium, particularly the heart valves. This study aimed to investigate culprit microorganisms among a cross-sectional cohort of IE patients, their associated factors, and to explore the potential relationship to the oral microbiome.

**Methods:**

In this observational study, we undertook a cross-sectional analysis of 392 medical records from patients diagnosed with IE. The primary outcome of this study was to analyse the association between the IE culprit microorganisms and the underlying anatomical types of IE (native valve (NVE), prosthetic valve (PVE), or cardiac device-related (CDE)). Secondary outcomes encompassed a comparative analysis of additional factors, including: the treatment approaches for IE, and the categorisation of blood cultures, extending to both genus and species levels. Additionally, we cross-referenced and compared the species-level identification of IE bacteraemia outcome measures with data from the expanded Human Oral Microbiome Database (eHOMD).

**Results:**

A culprit microorganism was identified in 299 (76.28%) case participants. Staphylococcal infections were the most common (*p* < 0.001), responsible for 130 (33.16%) hospitalisations. There were 277 (70.66%) cases of NVE, 104 (26.53%) cases of PVE, and 11 (2.81%) cases of CDE. The majority of PVE occurred on prosthetic aortic valves (78/104, 75%), of which 72 (93.5%) were surgical aortic valve replacements (SAVR), 6 (7.8%) were transcatheter aortic valve implants, and one transcatheter pulmonary valve implant. Overall, underlying anatomy (*p* = 0.042) as well as the treatment approaches for IE (*p* < 0.001) were significantly associated with IE culprit microorganisms. Cross-reference between IE bacteraemia outcomes with the eHOMD was observed in 267/392 (68.11%) cases.

**Conclusions:**

This study demonstrated that IE patients with a history of stroke, smoking, intravenous drug use, or dialysis were more likely to be infected with *Staphylococcus aureus*. CDE case participants and patients who had previous SAVR were most associated with *Staphylococcus epidermidis*. IE patients aged 78+ were more likely to develop enterococci IE than other age groups. Oral microorganisms indicated by the eHOMD are significantly observed in the IE population. Further research, through enhanced dental and medical collaboration, is required to correlate the presence of oral microbiota as causative factor for IE.

## 1 Introduction

Infective Endocarditis (IE) is a rare, life-threatening infection of the heart, particularly the heart valves, with multisystem effects (1). It affects up to 1:10,000 of the population, and has a high mortality rate that may reach up to 30% at 30 days post-infection (1). Culprit pathogens circulate in the blood and attach to cardiac tissue. These microorganisms can damage surrounding tissue and lead to vegetation and/or abscess formation, potentially causing life-threatening complications, including valvular insufficiency, heart failure and septic emboli to distal organs including the brain, lungs, spleen, and others (1).

Risk factors for IE encompass a broad range of medical and social determinants. Patients with prosthetic heart valves or implanted cardiac devices, including permanent pacemakers and cardioverter defibrillators, are at increased risk (1). Additionally, pre-existing conditions such as underlying structural heart disease, congenital heart disease, and various comorbidities significantly elevate the risk. Other factors, for example intravenous drug use (IVDU) and frequent contact with the healthcare system, also contribute to a heightened risk of developing IE (1). These factors collectively identify individuals as high-risk for IE, underscoring the importance of targeted preventive measures and vigilant monitoring.

Further, the oral microbiome represents an important source of recurrent bacteraemia, and recent literature has established that common dental procedures, frequently cause bacteraemia by oral commensals (2). Patients with active unstable periodontal disease are particularly at risk of bacteraemia even after routine tooth-brushing (2). Within the context of bacteraemia involving a pathogenic agent, the formation of infected vegetation can occur as the ultimate consequence of the complex interplay between the invading microorganisms and the immune response of the host. The involvement of oral pathogens in the onset of IE is attributed to the hematogenous dissemination of oral microorganisms (3). It has been reported that infections caused by the oral viridans group of streptococci (such as *Streptococcus mutans*, *Streptococcus mitis*, *Streptococcus sanguinis)* are responsible for nearly 20% of IE cases (3).

The oral microbiome is home to more than 700 bacterial species. Resources such as the expanded Human Oral Microbiome Database (eHOMD) offer a wealth of taxonomic and genomic information on oral microbes (3, 4). This data can be instrumental in analysing bacteraemia cases and investigating their potential origins in the oral microbiome (3, 4). The eHOMD Taxonomic Level page offers a detailed enumeration of taxa across the seven hierarchical levels of taxonomy: Domain, Phylum, Class, Order, Family, Genus, and Species. For instance, within the Genus level, it catalogues the 169 genera found in the HOMD, providing a count of species for each genus along with a link to the corresponding family (4). It utilises 16S rRNA RefSeq to enable comprehensive curated information on bacterial identification (4).

The management of IE usually necessitates the collaboration of a multidisciplinary team. Antimicrobial therapy (AT) is essential for all patients, while cardiovascular surgery may be advantageous for a specific subset (5). A positive blood culture is fundamental to diagnosing infections microbiologically. Obtaining three sets of blood cultures can identify 96%–98% of bacteraemia cases. Samples are routinely collected for culture prior to initiating antibiotic treatment in patients (5). Approximately 10% of patients with IE have blood cultures that yield no growth [blood culture negative endocarditis (BCNE)], leading to challenges in diagnosis. This phenomenon may be attributed to several factors, including the administration of antibiotics prior to taking blood cultures, infection with fastidious bacteria or fungi, or other conditions such as nonbacterial thrombotic endocarditis (5, 6).

The American Heart Association (AHA) and the European Society of Cardiology (ESC) reserve antibiotic prophylaxis (AP) for high-risk patients who undergo invasive dental procedures (7). This is in contrast to the National Institute for Health and Clinical Excellence (NICE guideline CG64 2008, and the 2015 update) in the United Kingdom. It advised the total discontinuation of AP to prevent IE in individuals with heart conditions who were scheduled for dental procedures (8). There remains uncertainty over the relationship between the oral microbiome and IE, and further insight is warranted.

We conducted an observational study to investigate culprit microorganisms among a cross-sectional cohort of IE patients, their associated factors, and to explore the potential relationship to the oral microbiome.

## 2 Materials and methods

### 2.1 Study design

This was an observational study focused on a cross-sectional cohort of adult patients diagnosed with IE (9). The study was approved as a service evaluation by King's College Hospital NHS Foundation Trust, Department of Cardiology (CV003-2022). Anonymised medical records of patients, who were admitted to the Cardiology Department at King's College Hospital (KCH) between December 2013 and February 2021, were assessed. This study was conducted in accordance with the Helsinki Declaration of 1975. Adult (≥18-years-old) patients with a diagnosis of definite or possible IE described by Modified Duke criteria were included (10).

### 2.2 Data collection

The electronic medical records of the included patients were analysed to extract anonymised data in relation to (i) demographics, (ii) comorbidities, (iii) other medical and social history factors, (iv) IE diagnosis confirmation (10), (v) bacteremia (at a genus and species level), (vi) underlying anatomy [native valve endocarditis (NVE), prosthetic valve endocarditis (PVE), cardiac device endocarditis (CDE)], (vii) type of IE treatment [AT, or surgical valve replacement (SVR)], and (viii) pre-existing cardiac conditions in adult patients known to increase the risk of developing IE (8, 11). All anonymised data were entered into a dedicated encrypted database. Access was restricted to data entry personnel and study investigators. Upon completion, the data were proofed for entry errors.

### 2.3 Outcome measures

The primary outcome of this study was to analyse the association between the IE culprit microorganisms and the underlying anatomical types of IE (native valve, prosthetic valve, or cardiac device-related). Secondary outcomes encompassed a comparative analysis of additional factors, including: the treatment approaches for IE, and the categorisation of blood cultures, extending to both genus and species levels. Additionally, we cross-referenced and compared the species-level identification of IE bacteraemia with data from the expanded Human Oral Microbiome Database (eHOMD, 16S rRNA RefSeq, Version 15.23).

### 2.4 Statistical analysis

Patient ages were categorised into four groups: 18–37, 38–57, 58–77 and 78+. Culprit microorganisms were categorised into groups by genus and analysed for trends. *Staphylococcus*, *Streptococcus* and *Enterococcus* were genus groups of focus, as they are cumulatively responsible for up to 90% of all IE (1). Microorganisms not belonging to these genus groups were defined as “Other”. In cases of BCNE the term “None” was used. For genus groups returning an association with analysed factors, species within the genus were then investigated.

A normality test was conducted using Q-Q Plots and the Kolmogorov–Smirnov test. The analysis of the microorganisms responsible IE and their association with specific factors–namely, the most prevalent culprits, the underlying anatomical types of IE, and the IE treatment methods–was carried out using the *χ*^2^ test (*p* < 0.05). Descriptive statistics were employed to identify and compare the infection characteristics across different groups of microorganisms. IE culprit microbiology results were cross-referenced with the eHOMD at a descriptive level (12). Analyses were performed IBM SPSS Statistics [Version 29.0.2.0 (20), IBM Corp, New York, United States, 2023].

## 3 Results

### 3.1 Baseline characteristics

A total of 392 cases were included (Table 1). The mean age of patients with IE was 61.4 years (range 20–93), comprising predominantly males (264/392, 67.35%). The ethnic demographic of this cross-sectional cohort was 63.78% (*n* = 250) white, 12.50% (*n* = 49) black, 1.79% (*n* = 7) mixed, 5.87% (*n* = 23) Asian, 4.08% (*n* = 16) other ethnicity and 11.99% (*n* = 47) with null information. Eleven associated comorbidities were identified including Coronary Heart Disease (CHD), Heart Failure (HF), Atrial Fibrillation (AF), Chronic Obstructive Pulmonary Disease (COPD), Diabetes Mellitus (DM), Chronic Kidney Disease (CKD), Stroke (ST), Cancer (CA), Hypertension (HTN), Chronic Liver Disease (CLD) and Hyperlipidaemia (HLD). Comorbidities identified in this population are summarised in Table 1. Each patient had at least two comorbidities (*n* = 908), with the most common conditions being HTN (*n* = 232, 59.18%), CA (*n* = 112, 28.57%) and HLD (*n* = 99, 25.26%). Other associated factors for IE identified in this patient population included intravenous drug use (IVDU) (*n* = 34, 8.7%), smoking (*n* = 27, 6.9%), and dialysis (*n* = 20, 5.1%).

**Table 1:** Demographic and descriptive data for the infective endocarditis patient population.

Patient features	IE diagnosis confirmation		All
	Definite IE	Possible IE	
Age, y			
18–37	31 (83.8)	6 (16.2)	37 (100)
38–57	83 (75.5)	27 (24.5)	110 (100)
58–77	119 (69.6)	52 (30.4)	171 (100)
78+	56 (75.7)	18 (24.3)	74 (100)
Sex			
Male	202 (76.5)	62 (23.5)	264 (100)
Female	87 (68)	41 (32)	128 (100)
Ethnicity			
White British	169 (75.4)	55 (24.6)	224 (100)
White other	18 (69.2)	8 (30.8)	26 (100)
Black	35 (71.4)	14 (28.6)	49 (100)
Asian	16 (69.6)	7 (30.4)	23 (100)
Mixed	5 (71.4)	2 (28.6)	7 (100)
Other	11 (68.8)	5 (31.2)	16 (100)
Null	35 (74.5)	12 (25.5)	47 (100)
Comorbidities			
CHD	32 (61.5)	20 (38.5)	52 (100)
HF	61 (71.8)	24 (28.2)	85 (100)
AF	62 (77.5)	18 (22.5)	80 (100)
COPD	33 (80.5)	8 (18.5)	41 (100)
DM	10 (66.7)	5 (33.3)	15 (100)
CKD	57 (70.4)	24 (29.6)	81 (100)
ST	74 (77.1)	22 (22.9)	96 (100)
CA	73 (65.2)	39 (34.8)	112 (100)
HTN	162 (69.8)	70 (30.2)	232 (100)
CLD	12 (80)	3 (20)	15 (100)
HLD	70 (70.7)	29 (29.3)	99 (100)
Other factors			
IVDU	30 (88.2)	4 (11.8)	34 (100)
Smoking	25 (92.6)	2 (7.4)	27 (100)
Dialysis	12 (60)	8 (40)	20 (100)
Bacteraemia (Genus)			
Staphylococcus	106 (81.5)	24 (28.5)	130 (100)
Streptococcus	76 (83.5)	15 (26.5)	91 (100)
Enterococcus	39 (88.6)	5 (11.4)	44 (100)
Other	28 (82.4)	6 (17.6)	34 (100)
None (BCNE)	40 (43)	53 (57)	93 (100)
Underlying anatomy			
NVE	203 (73.3)	74 (26.7)	277 (100)
PVE	77 (74)	27 (26)	104 (100)
CDE	9 (81.8)	2 (18.2)	11 (100)
Treatment for IE			
SVR	100 (85.5)	17 (14.5)	117 (100)
AT	189 (68.7)	86 (31.3)	275 (100)

Values are *n* of patients or procedures (%).

CHD, coronary heart disease; HF, heart failure; AF, atrial fibrillation; COPD, chronic obstructive pulmonary disease; DM, diabetes mellitus; CKD, chronic kidney disease; ST, stroke; CA, cancer; HTN, hypertension; CLD, chronic liver disease; HLD, hyperlipidaemia; IVDU, intravenous drug use; NVE, native valve endocarditis; PVE, prosthetic valve endocarditis; CDE, cardiac device endocarditis; IE, infective endocarditis; SVR, surgical valve replacement; AT, antimicrobial therapy.

In terms of the underlying anatomy, there were 277 (70.66%) cases of NVE, 104 (26.53%) cases of PVE and 11 (2.81%) cases of CDE. Of PVE patients, the majority had SVR (*n* = 97, 93.3%), rather than transcatheter implanted valves (*n* = 7, 6.7%). The majority of PVE occurred on prosthetic aortic valves (78/104, 75%), of which 72 were surgical aortic valve replacements (SAVR) and 6 were transcatheter aortic valve implants (TAVI). Patients affected at the site of either an implantable cardioverter defibrillator (ICD) or permanent pacemaker (PPM) constituted CDE. Valve regurgitation was observed in higher proportion in Definite IE (*n* = 49, 86%) patients in comparison to Possible IE (*n* = 8, 14%). The majority of patients underwent medical treatment (*n* = 275, 70.2%), rather than surgical management (*n* = 117, 29.8%).

### 3.2 Microbiological outcomes

The most abundant IE culprit microorganism corresponded to the *Staphylococcus* (33.16%)*,* followed by *Streptococcus* (23.01%)*,* and *Enterococcus* (11.22%)*,* genera [*χ*^2^(4) = 78.944, *p* < 0.001]. Other microorganisms (8.93%) were mainly characterised by HACEK organisms [*Haemophilus influenzae (n = 2)*, *Aggregatibacter aphrophilus (n = 3)*, *Aggregatibacter actinomycetemcomitans (n = 2)*] (13). There were 93/392 (23.72%) cases of BCNE.

The most prevalent species was *Staphylococcus aureus* (*n* = 93, 23.72%). The full range of culprit microorganisms is presented in Table 2.

**Table 2:** Culprit microorganisms derived from the IE patient population.

Culprit microorganism	Number of cases (%)
*Staphylococcus (S.)* species	130 (33.16%)
*S. aureus*	93 (23.72%)
*S. epidermidis*	22 (5.61%)
*S. warneri*	9 (2.30%)
*S. hominis*	2 (0.51%)
*S. capitis*	1 (0.26%)
*S. lugdunensis*	1 (0.26%)
*S. pettenkoferi*	1 (0.26%)
*S. haemolyticus*	1 (0.26%)
*Streptococcus (Strep.)* species	90 (23.01%)
*Strep. mitis*	23 (5.87%)
*Strep. gallolyticus*	17 (4.34%)
*Strep. agalactiae*	14 (3.57%)
*Strep. sanguinis*	7 (1.79%)
*Strep. salivarius*	6 (1.53%)
*Strep. anginosus*	4 (1.02%)
*Strep. pneumoniae*	4 (1.02%)
*Strep. mutans*	3 (0.77%)
*Strep. gondonii*	3 (0.77%)
*Strep. infantarius*	2 (0.51%)
*Strep. cristatus*	1 (0.26%)
*Strep. parasanguinis*	1 (0.26%)
*Strep. vestibularis*	1 (0.26%)
*Strep. constellatus*	1 (0.26%)
*Strep. oralis*	1 (0.26%)
*Strep. dysgalactiae*	1 (0.26%)
*Strep. canis*	1 (0.26%)
*Enterococcus (Ent.)* species	44 (11.22%)
*Ent. faecalis*	40 (10.20%)
*Ent. faecium*	4 (1.02%)
*Other* species	35 (8.93%)
*Aggregatibacter aphrophilus*	3 (0.77%)
*Haemophilus influenzae*	2 (0.51%)
*Aggregatibacter actinomycetemcomitans*	2 (0.51%)
*Candida albicans*	2 (0.51%)
*Gemella haemolysans*	2 (0.51%)
*Abiotrophia defectiva*	2 (0.51%)
*Corynebacterium amycolatum*	2 (0.51%)
*Granulicatella adiacens*	2 (0.51%)
*Kytococcus schroeteri*	2 (0.51%)
*Neisseria elongata*	2 (0.51%)
*Aerococcus viridans*	1 (0.26%)
*Gemella sanguinis*	1 (0.26%)
*Corynebacterium diphtheriae*	1 (0.26%)
*Corynebacterium propinquum*	1 (0.26%)
*Escherichia coli*	1 (0.26%)
*Klebsiella oxytoca*	1 (0.26%)
*Gemella morbillorum*	1 (0.26%)
*Actinomyces meyeri*	1 (0.26%)
*Neisseria gonorrhoeae*	1 (0.26%)
*Rothia dentocariosa*	1 (0.26%)
*Rothia aeria*	1 (0.26%)
*Serratia marcescens*	1 (0.26%)
*Tropheryma whipplei*	1 (0.26%)
*Leuconostoc sp.*	1 (0.26%)
None (BCNE)	93 (23.72%)

A significant association was observed between the microorganisms responsible for IE and the underlying anatomical types of IE [*χ*^2^(4) = 9.926, *p* = 0.042]. For patients with NVE, *Staphylococcus* (86/277, 31.04%) and *Streptococcus* (70/277, 25.27%) species were both more prevalent than *Enterococcus* (29/277, 10.46%) and Other organisms (23/277, 8.30%). BCNE (69/277, 24.91%) was also more prevalent than *Enterococcus* and Other organisms. In PVE patients, although *S. aureus* remained the leading cause of *Staphylococcal* infection (15/36, 41.66%), *S. epidermidis* (11/36, 30.55%) and *S. warneri* (7/36, 19.44%) were greater causes in the PVE population than the NVE population (8.14% and 0% respectively). Patients who have had a TAVI had a higher frequency to develop *Enterococcus* endocarditis (3/6, 50%) than those who have had a SAVR (8/72, 11.11%). The most common culprit species of endocarditis in the TAVI population was *Enterococcus faecalis*. *Staphylococcus epidermidis* was the most represented species in the SAVR population (9/72, 12.5%), while *Strep. mitis* (4/20, 20%) and *Strep. gallolyticus* (4/20, 20%) were the most represented *Streptococcus* species in patients with SAVR. IE patients were more likely to have *Staphylococcus* infection than another genus, if they had the associated factors of stroke (42/96, 43.75%), smoking (15/27, 55.55%), intravenous drug use (IVDU) (19/34, 55.88%) or dialysis (8/20, 40%), of which *Staphylococcus aureus* was the most represented species for each of these patient groups. CDE cases were most associated with *Staphylococcus epidermidis* (4/11, 36.36%). There were two cases (18.18%) of BCNE affecting patients with cardiac devices; the rest were all caused by *Staphylococci*.

IE patients who were in the eldest category (aged 78–97 years) were more likely to have been infected with *Enterococci* (18/44, 24.32%) compared to those in the other age categories (26/318, 8.18%). *Enterococci* affected older ages compared to *Staphylococci*, Other and None genus organisms. The mean (±SD) age of patients with *Staphylococcal* infection was 59.59 (±16.47) years, *Streptococcal* was 63.60 (±15.88) years, *Enterococcal* was 68.82 (±15.87) years, Other genus was 54.26 (±20.21) years and No genus was 60.83 (±14.49) years. Additionally, a significant association was found between the types of microorganisms causing IE and the treatment strategies implemented for IE. Antibiotic therapy emerged as the predominant treatment strategy for IE [*χ*^2^(2) = 15.154, *p* < 0.001].

A secondary descriptive comparison sub-analysis to understand the cross-reference between IE culprit bacteraemia microbiological outcomes with the oral microbiome was performed. The eHOMD, provides comprehensive and curated information on almost 800 oral bacterial species reliably identified in the oral cavity (eHOMD, 16S rRNA RefSeq, Version 15.23) (12). This cross-reference sub-analysis showed that 68.11% (267/392) of patients in the cross-sectional cohort tested positive for bacterial species that have been reliably identified in the oral microbiome via the eHOMD dataabase (Table 3).

**Table 3:** Cross-referenced descriptive results by genus and species level between IE microbiological outcomes and the oral microbiome via eHOMD.

Bacteraemia (*n* = number of specified microorganism identified in population)	eHOMD, expanded human Oral Microbiome Database	
	Present in eHOMD (+)	Absent in eHOMD (-)
*Staphylococcus* (130)	*Staphylococcus aureus (93)*	Null
	*Staphylococcus epidermidis (22)*	
	*Staphylococcus warneri (9)*	
	*Staphylococcus hominis (2)*	
	*Staphylococcus capitis (1)*	
	*Staphylococcus lugdunensis (1)*	
	*Staphylococcus pettenkoferi (1)*	
	*Staphylococcus haemolyticus (1)*	
*Streptococcus* (90)	*Streptococcus mitis (23)*	*Streptococcus gallolyticus (17)*
	*Streptococcus agalactiae (14)*	*Streptococcus Infantarius (2)*
	*Streptococcus sanguinis (7)*	*Streptococcus Canis (1)*
	*Streptococcus salivarius (6)*	*Streptococcus Dysgalactiae (1)*
	*Streptococcus anginosus (4)*	
	*Streptococcus pneumoniae (4)*	* *
	*Streptococcus mutans (3)*	* *
	*Streptococcus gordonii (3)*	* *
	*Streptococcus cristatus (1)*	* *
	*Streptococcus oralis (1)*	* *
	*Streptococcus vestibularis (1)*	* *
	*Streptococcus parasanguinis (1)*	* *
	*Streptococcus constellatus (1)*	* *
*Enterococcus* (44)	*Enterococcus faecalis (40)*	*Enterococcus faecium (4)*
Other (35)	*Aggregatibacter aphrophilus (3)*	*Kytococcus schroeteri (2)*
	*Neisseria elongate (2)*	*Candida albicans (2)*
	*Abiotrophia defective (2)*	*Klebsiella oxytoca (1)*
	*Corynebacterium amycolatum (2)*	*Tropheryma whipplei (1)*
	*Granulicatella adiacens (2)*	*Leuconostoc sp. (1)*
	*Gemella haemolysans (2)*	* *
	*Haemophilus influenzae (2)*	* *
	*Aggregatibacter actinomycetemcomitans (2)*	* *
	*Gemella morbillorum (1)*	* *
	*Corynebacterium diphtheriae (1)*	* *
	*Serratia marcescens (1)*	* *
	*Rothia aeria (1)*	* *
	*Rothia dentocariosa (1)*	* *
	*Corynebacterium propinquum (1)*	* *
	*Actinomyces meyeri (1)*	* *
	*Escherichia coli (1)*	* *
	*Neisseria gonorrhoeae (1)*	* *
	*Aerococcus viridans (1)*	* *
	*Gemella sanguinis (1)*	* *

## 4 Discussion

### 4.1 Main findings

The mean age of infection in the present studied population IE was 61.4 years old, consistent with other studies in the literature (14, 15). Men were more affected than women, in a 2:1 ratio, although the spread of culprit organisms did not differ between the two sexes. This is consistent with other literature that reports the male:female ratio of IE between 2:1 and 9:1 (16). Studies attribute the differences in infection rate to a variety of factors including variable comorbidities, treatment biases, or inherent physiologic differences (17).

*Staphylococcus aureus* was a leading culprit of IE among patients with a history of stroke, smokers, IVDU or on dialysis. It has been reported that the association between *S. aureus* and stroke is bidirectional, as both can act as a risk factor for the other; stroke induces immunosuppression which increases the risk of infection and *S. aureus* has demonstrated the ability to form vegetations on heart valves, which can embolise to cause cerebral infarctions (18, 19). Cigarette smoking heightens bacterial virulence, promoting *S. aureus* to a virulence profile associated with persistent infection, causing increased biofilm formation, invasiveness and intracellular persistence (20). Literature highlights *S. aureus* as an especially prevalent culprit organism among IVDU patients (21). This is supported by our findings where all IVDU status IE patients with *Staphylococci* infection were caused by *S. aureus*. Improved education and implementation of infection control and prevention measures from healthcare providers to service users will assist patients most at risk. *Staphylococci* normally reside on the skin without causing issues; however, it can cause infection when there is any disruption of the skin barrier, for example with IVDU or dialysis. These bacteria are highly contagious and can easily spread through contact. Good hand hygiene precautions, cross-contamination awareness and thorough sterilisation of medical equipment are important preventative measures.

Cardiac devices, such as an ICD or PPM, can act as attachment sites for circulating bacteria. Coagulase-negative *Staphylococci* represent the majority of culprit organisms responsible for CDE, consistent with other reports (22), although, in recent years, the prevalence of *Staphylococcus aureus* among CDE patients appears to be increasing (23). The reason for *S. aureus* increasing prevalence among CDE cases is not well described in the literature, nonetheless, this transition is concerning as *S. aureus* is associated with a worse prognosis than *S. epidermidis* (24).

*Streptococci* are typically located in the oral cavity and respiratory tract, contributing approximately 20% of the global IE burden, with greater incidence in resource-limited and developing regions (25). The largest group of these bacteria, viridans group *Streptococci* (VGS), are commonly found among those with poor dental health and conditions such as dental caries and periodontal disease. *Strep. sanguinis* is particularly important as it is one of the most common causative agents of IE, comprising approximately 30% of VGS cases (26, 27). Normally within the oral environment, *Strep. sanguinis* is a primary coloniser of the tooth surface, protecting the human host against the damaging effects of another microorganism, *Strep. mutans*, which is responsible for tooth decay and caries (28). Interestingly, for this cross-sectional cohort of IE patients, the frequency of infection from *Strep. sanguinis* was far lower than expected (*n* = 7, 1.79%). Protective measures against *Streptococcal* infection might include regular toothbrushing, frequent professional dental cleaning and infection control. Antibiotic prophylaxis before dental procedures might offer protection to patients at high risk of IE. In the UK, the National Institute for Health and Care Excellence (NICE) concluded that there is insufficient evidence to recommend routine antibiotic prophylaxis for dental treatment for at-risk patients, despite incidence rates of IE observing an increase since this recommendation (29). Further evidence and studies regarding the efficacy of antibiotic prophylaxis are needed (30).

This study reports that the origin of organisms identified from microbiology are greatly cross-referenced with the oral microbiome, via eHOMD cross-referencing. However, this is not definitive evidence that the route of infection was from the oral cavity, as many organisms have various distinct origins, with various modes of entry into the bloodstream. For example, *Staphylococci* are ubiquitously found in normal skin flora, whilst *Enterococci* originate from the gastrointestinal flora, although organisms from both genera have been identified in the oral microbiome, thereby offering an alternative bacterial port of entry to consider (12). Furthermore, there were two cases of fungal IE due to *Candida albicans* infection and their presence in the oral cavity is well documented (31, 32). Collectively, these findings underscore the critical importance of rigorous oral hygiene and overall oral health for patients at risk for IE. As a result, it is recommended that dentists collaboratively integrate with the multidisciplinary medical team to ensure that IE patients receive a comprehensive dental examination and a detailed dental history at the time of admission, particularly before undergoing cardiac surgery. Whenever feasible, and the patient is stable enough to be treated in a dental chair, these examinations/management should be conducted under the vigilant oversight and guidance of the medical team.

*Enterococci* were a leading culprit of IE among patients with advancing age and have undergone TAVI procedure. The prevalence of IE in the elderly population is steadily increasing, notably due to the rising number of invasive procedures and cardiac devices implanted in these patients. Our findings are consistent with other reports of *Enterococci* as the most common culprits of TAVI, followed by *S. aureus* (28, 33). Femoral access for TAVI could be an independent risk factor for *Enterococci* infection due to its close proximity to gastrointestinal output. Improving our knowledge of the aetiology of IE in the elderly is essential to disentangle any confounding factors and whether this is an issue of co-linearity or independent risk. *E. faecalis* infection can occur through faecal-oral transmission and spread can be mitigated through effective hygiene practices. *Enterococci* have become a major cause of nosocomial infection (34). Improperly cleaned catheters, dialysis ports, and other medical devices can also carry *E. faecalis*. Thus, people who undergo organ transplants, kidney dialysis, or cancer treatment are at increased risk for developing infections due to immunosuppression or contamination through their catheters. Patients who undergo TAVI instead of SAVR are typically already at high-risk (35), and therefore, could be more susceptible to *E. faecalis* infection due to other factors, rather than TAVI as an independent cause. However, there are various confounding factors regarding the relationship between TAVI and *E. faecalis* infection, whereby future research will be crucial in identifying methods to potentially improve patient outcomes who have had a TAVI.

BCNE accounts for approximately 20% of IE and can be caused by a variety of microorganisms, including *Coxiella burnetii*, *Bartonella spp*., *Brucella spp*., *Tropheryma whipplei*, *Mycoplasma spp*., *Legionella spp*., and non-candida fungi (6). Pre-emptive administration of antibiotic therapy is the most prevalent reason for BCNE (6). Approximately half of patients with IE undergo early surgery and 16S ribosomal ribonucleic acid (rRNA) polymerase chain reaction (PCR) of excised tissue can be vitally important to secure a diagnosis (6). 16S rRNA sequencing represents a form of next generation sequencing (NGS), which yields greater sensitivity than traditional culture methods and involves library preparation, fragmenting DNA/RNA into clusters, sequencing, and reassembling to form a genomic sequence for analysis (36). Precision medicine (e.g., pharmacogenomics) is likely to become a key tool in improving outcomes from BCNE and will contribute to an improved aetiological diagnosis and prognosis moving forwards, but this is yet to become a routine element of clinical practice.

### 4.2 Limitations

Although our patient population is primarily derived from the London Boroughs of Lambeth, Southwark and Bromley, the department operates as a tertiary referral centre for millions. In order to reduce selection bias, a large patient population was included. The retrospective nature of this study provides inherent limitations when compared to prospective longitudinal studies. Prospective studies, with an analysis of the patient's oral health status, would provide a more robust insight into the association between the oral microbiome and IE. Confounding factors such as the clinical variation of periodontal disease among the study population resulted in difficulty in drawing firm conclusions. Furthermore, due to the nature of the study, we were only able to cross-reference culprit microorganisms of IE and the oral microbiome using the eHOMD. Whilst the eHOMD is a reliable database detailing bacteria taxonomy that has been identified in the oral microbiome, these bacterial species could have also instigated disease through other routes and future prospective studies are required by a multidisciplinary team of dentists and doctors to clarify any association.

## 5 Conclusions

Infective endocarditis patients with a history of stroke, smoking, IVDU or dialysis were more likely to be infected with *Staphylococcus aureus*. CDE cases and patients who had previously undergone SAVR were most associated with *Staphylococcus epidermidis*. Infective endocarditis patients who were elderly were more likely to have been infected with *Enterococcus faecalis*. Patients who underwent TAVI instead of SAVR were more likely to have been infected with *Enterococcus faecalis*. From the study population, 68.11% of cases were caused by organisms that have been identified in the oral microbiome via the eHOMD. Nevertheless, additional research is needed to better understand the connection between the oral microbiome and infective endocarditis.

## Data Availability

The datasets for this article are not publicly available. Requests to access the datasets should be directed to the corresponding author.
